# Succession of Composition and Function of Soil Bacterial Communities During Key Rice Growth Stages

**DOI:** 10.3389/fmicb.2019.00421

**Published:** 2019-03-11

**Authors:** Wenhui Wang, Xue Luo, Yang Chen, Xianfeng Ye, Hui Wang, Zhe Cao, Wei Ran, Zhongli Cui

**Affiliations:** ^1^Key Laboratory of Soil Environment and Pollution Remediation, Institute of Soil Science, Chinese Academy of Sciences, Nanjing, China; ^2^Key Laboratory of Agricultural Environmental Microbiology of the Ministry of Agriculture, Nanjing Agricultural University, Nanjing, China; ^3^Department of Plant Sciences, Crop Development Centre, University of Saskatchewan, Saskatoon, SK, Canada; ^4^College of Resources and Environmental Sciences, Nanjing Agricultural University, Nanjing, China

**Keywords:** bacterial community, bacterial function, NPK fertilizers, molecular ecology network, rice growth stages

## Abstract

Elucidating the succession of soil microbial communities and microbial functions at key plant growth stages is a major goal of microbial ecology research. In this study, we investigated the succession of soil bacteria during four fertilizer treatments (control, NPK, NPK + pig manure, and NPK + straw) and at three crucial rice growth stages (tillering, heading, and ripening) in paddy soil from a rice-wheat cropping system over a 10-year period. The results showed that the bacterial community and function composition of the control treatment was significantly different from that of the other treatments with NPK fertilizers, and S1 from others stages (ANOSIM, *p* < 0.05). The application of pig manure could reduce the effects of applying NPK fertilizers on bacterial communities in heading and ripening stages, but the effects of straw returning is not obvious. Variance partitioning analyses (VPA) suggested that pH, OM, and AK appeared to be key factors responsible for the microbial community changes observed in all the treatments or stages. The correlation results showed the bacterial families different between S1 and other stages such as *Micromonosporaceae*, *Nocardioidaceae*, *Gaiellaceae*, and *Anaerolineaceae* etc., were correlated with bacterial KEGG metabolic pathways. In addition, the topological of the soil bacterial community network with more nodes, links and higher Maximal degree at the heading stage and maintained relatively similar topological structures at the heading and ripening stages. However, the topological of the functional networks at the ripening stage were a small yet complicated co-occurring network with 209 nodes, 789 links, higher Average connectivity (avgK), and Maximal degree. These results suggest an obvious succession of soil bacteria and bacterial function at the key rice growth stages, but the topological of functional network structure of bacteria changes a little in the early and middle stages of rice, while its changes significantly in the ripening stage of rice growth.

## Introduction

The rice-wheat cropping system is the main grain production system in the Yangtze River Basin in China (Wang et al., 2016a), and soil acidification, continuous cropping obstacles and soil-borne diseases are the major issues affecting rice-wheat production in this area. Previous case studies showed that the application of organic amendments, such as pig manure and straw, could either improve the soil community structure (Blanchet et al., 2016) or alleviate soil acidification (Sun et al., 2015) and continuous cropping obstacles (Zhao et al., 2016). Fertilization can also significantly change the community structure of soil microorganisms. A proper understanding of the mutual relationship between the soil microbial structure and fertilizer management at different phenological stages of rice growth will aid the selection of appropriate and efficient fertilizers for different phenological stages of rice growth.

Due to the rapid responses of bacteria to changes in the soil environment, bacterial communities were selected as early bio-indicators of soil quality (Chen et al., 2017). Previous studies revealed that root exudates have a significant impact on the soil microbial community (Shi et al., 2013). In addition, the interactions between the rice root and soil microorganisms usually induce growth promotion and disease suppression (Edwards et al., 2015). But at different growth stages, crop root exudates commonly undergo dynamic changes (Li et al., 2016). Several studies using different methods for testing diversity reported that the soil bacterial and functional gene communities show obvious differences among different plant growth stages (Noll et al., 2005; Rui et al., 2009; Krause et al., 2010; Edwards et al., 2015; Li et al., 2016; Wang et al., 2016a). Soil microbial communities also play an important role in the maintenance of ecosystem function and sustainability (Paul and Clark, 1989). Therefore, it is essential to explore the internal dynamics among soil bacteria, the functional gene community, and the fertilizer and plant growth stage, which are important factors that influence the ecosystem function and sustainability of the cropping system.

Molecular ecology network analysis has been used as a powerful tool to understand soil microbial ecosystems and complex microbial assemblages (Zhou et al., 2011; Deng et al., 2012). The co-occurrence (positive or negative interaction) among OTUs/genera can be defined by a molecular ecological network obtained using random matrix theory-based methods (Zhou et al., 2011; Deng et al., 2012). These visual network structures provide detailed perspectives on microbial assemblages (Shi et al., 2016). The potential microbe-microbe interactions display significant changes among different growth stages of upland plants, and the complexity of the microbial network increases during the growth of the *Avena fatua* (Shi et al., 2016). In addition, based on the 16S rRNA gene sequences, phylogenetic investigation of communities by reconstruction of unobserved states (PICRUSt) has been widely used to predict the functional capabilities of microbial communities (Langille et al., 2013). In this study, we explored the soil microbial and microbial function networks of a rice-wheat cropping system at different rice growth stages.

Four fertilizer treatments (control, NPK, NPK + manure, and NPK + straw) were applied to a rice field and paddy soil with a distribution of three rice growth stages (tillering, heading, and ripening), and samples were collected for the analysis of the bacterial community structure. The objectives of this study were to (1) understand the effects of fertilizers and rice growth stages on the soil bacterial community structure and bacterial metabolic functions, (2) explore the key soil environmental variables that strongly affected the succession of the bacterial community during the paddy stages in a rice-wheat cropping system, and (3) understand the variations between the topological shift of the soil bacterial networks and that of the metabolic functional networks during the rice growth stages.

## Materials and Methods

### Field Description and Experimental Design

The experiments were performed in a paddy field in Changzhou, Jintan, China (31°39′N, 119°28′E), during rice-wheat rotation in 2005. The local climatic conditions is a northern subtropical monsoon with an annual precipitation of 1063 mm and an average annual temperature of 15.3°C. Based on the FAO soil classification system, the field soil is classified as stagnic anthrosols. A randomized block design with three replicates (each plot consisted of 8 m × 5 m) was used in the field experiment. Four common fertilization treatments were designed: A, control treatment without fertilizers; B, 100% NPK chemical fertilizer treatment (based on the conventional dosage for this region, namely, 300 kg/ha urea, 120 kg/ha P_2_O_5_, and 100 kg/ha K_2_O); C, 50% NPK fertilizer treatment plus 6,000 kg/ha pig manure; and D, 100% NPK fertilizer treatment plus crop straw. All other fertilizers and crop straw were applied as basal fertilizers before planting, whereas N fertilizers were used as basal and supplementary fertilizers (basal fertilizers: tillering supplementary fertilizers: panicle supplementary fertilizers = 4:3:3).

### Soil Sampling and Analysis of Soil Properties

In 2015, the soil was sampled at three rice-growing stages, namely, at the tillering stage in July (14 days after seed sowing), the booting stage in September (56 days after seed sowing) and the ripening stage in October (98 days after seed sowing). A randomized block design with three replicates (each plot consisted of 8 m × 5 m) was used in the field experiment. For each plot, Soil samples (diameter of 2.5 cm and depth of 0–20 cm) from seven to 11 randomly selected locations were pooled to obtain one biological replicate. After the removal of rice roots and stones, the pooled soil sample was placed in a sterile plastic bag in an ice box until transport to a laboratory for further sample storage or chemical analyses (Wang et al., 2016c).

### Soil DNA Extraction and High-Throughput Sequencing

The total soil DNA from 0.5 g of soil was extracted using a fast PowerSoil DNA Isolation Kit (MP Biomedicals, Santa Ana, CA, United States) according to the manufacturer’s instructions. The bacterial V4-V5 hypervariable region of the 16S rRNA gene was amplified by PCR using the primers 515F (5′-CCTACGGGAGGCAGCAG-3′) and 907R (5′-TTACCGCGGCTGCTGGC-3′). PCR reactions were performed in triplicate 20 μL mixture containing 4 μL of 5 × FastPfu Buffer, 2 μL of 2.5 mM dNTPs, 0.8 μL of each primer (5 μM), 0.4 μL of FastPfu Polymerase (Transgen, China), and 10 ng of template DNA. The PCR products from the soil samples were sent out for pyrosequencing using an Illumina MiSeq PE300 (Illumina Inc., San Diego, CA, United States) at the Majorbio Bioinformatics Technology Co., Ltd. (Shanghai, China) (Wang et al., 2016c). The Trimmomatic (Bolger et al., 2014) and flash (Magoč and Salzberg, 2011) programmes were used to process the V4-V5 tagged sequences. The 16S rRNA operational taxonomic units (OTUs) with an identical cut-off of 0.97 were processed using the Usearch software platform (version 7.1)^1^ (Edgar et al., 2011), and chimeric OTUs were then removed using the Uchime (version 4.2.40) method (Edgar, 2013). A subset of 47,847 sequences per sample was selected for downstream analyses. The raw sequences are available through the NCBI Sequence Read Archive (Accession No. SUB3055082).

### Statistical Analyses

The values of richness estimators [coverage estimator (Ace) and abundance-based (Chao1)] and diversity indices (Shannon and Simpson) were determined using Mothur (Schloss et al., 2011). Principal coordinate analysis (PCoA), analysis of similarities (ANOSIM), redundancy analysis (RDA), Monte Carlo permutations and variance partitioning analyses (VPA) were performed using R (Version 3.1.2, vegan package). The bacterial metabolic function profiles (KEGG and COG) were generated using PICRUSt. Comparisons of the top 100 bacterial families and bacterial metabolic functions among the different groups (fertilizer treatments and growth stages) were performed using STAMP (Parks et al., 2014). The correlations between bacterial abundance (at the family level, forward selected by STAMP) and PICRUSt-generated KEGG metabolic pathway profiles were plotted using R (version 3.1.2, corrplot2 package). A network analysis was performed using the Molecular Ecological Network Analyses Pipeline^2^ (Zhou et al., 2010, 2011; Deng et al., 2012). The “output for Cytoscape visualization” was run in the “greedy modularity optimization mode,” and the files for network graph visualization were generated using a Gephi interactive platform (Bastian et al., 2009). The other statistical analyses were conducted using SPSS 13.0.

## Results

### Soil Physicochemical Characteristics

The soil organic material (OM), total K (TK), available N (AN), available P (AP), and pH were significantly different (*p* < 0.05) among the four fertilizer treatments at all three rice growth stages (Tables 1, 2). In addition, the soil pH, available K, and OM showed obvious increase or decrease (*p* < 0.05) at the three different stages (Table 1). The soil pH, TP, and AN were more likely to show changes among different fertilization treatments and rice growth stages than the other soil physicochemical characteristics (Table 2, Two-way ANOVA, *p* < 0.001). At all stages, particularly the ripening stage (S3), the control without fertilizers (A) had a higher soil pH but lower contents of OM, TP, TK, and AP compared with the other three fertilization treatments. Additionally, the addition of pig manure to the NPK treatment (C) significantly increased (*p* < 0.05) the OM and AP contents.

**Table 1:** Chemical properties at different soil stages.

Field		OM		TN		TP		TK		AN		AP		AK		pH	
	
		Mean	*SD*	Mean	*SD*	Mean	*SD*	Mean	*SD*	Mean	*SD*	Mean	*SD*	Mean	*SD*	Mean	*SD*
S1	A	26.80d	0.92	1.85ab	0.10	0.79abcd	0.12	13.13a	0.13	250.20ab	12.45	15.88bcd	0.20	116.33ab	10.79	7.76a	0.02
	B	27.76d	3.01	1.71b	0.04	0.79abcd	0.21	12.27ab	0.38	237.18ab	25.24	12.89de	2.63	107.67b	1.53	7.24b	0.15
	C	32.78abc	0.46	2.03a	0.29	0.97a	0.02	11.28bc	0.23	258.01a	31.42	33.17a	4.53	141.33a	18.23	7.17bc	0.09
	D	29.84cd	1.15	1.90ab	0.04	0.81abcd	0.12	11.14bc	1.06	226.72abc	16.73	20.14bc	2.75	141.67a	19.40	7.04bc	0.05
S2	A	28.91cd	2.26	1.72b	0.02	0.58de	0.03	10.59cd	0.40	178.49c	15.94	8.56ef	1.37	98.00b	13.45	7.27b	0.09
	B	30.25bcd	2.24	1.86ab	0.09	0.68cde	0.05	9.76d	0.35	196.51bc	16.66	14.76bcde	2.77	94.33b	19.55	7.03bc	0.15
	C	36.23a	1.12	2.14a	0.05	0.93ab	0.07	9.60d	0.43	225.25abc	13.73	30.26a	3.32	99.33b	8.33	6.90c	0.05
	D	33.18abc	2.48	2.06a	0.08	0.71bcde	0.04	10.66cd	0.80	237.27ab	24.06	19.67bcd	4.17	106.67b	17.62	6.52d	0.14
S3	A	27.16d	1.56	1.63b	0.03	0.50e	0.02	9.66d	0.50	184.16c	10.42	5.00f	0.69	99.33b	2.08	7.60a	0.09
	B	31.48bcd	1.03	1.84ab	0.12	0.67cde	0.03	13.30a	0.65	215.38abc	16.70	15.05bcde	1.84	89.67b	7.09	7.18bc	0.12
	C	34.78ab	0.96	2.03a	0.02	0.86abc	0.02	13.58a	0.06	240.91ab	19.98	21.43b	0.23	104.33b	1.15	7.28b	0.02
	D	32.66abc	1.10	1.92ab	0.08	0.70bcde	0.08	13.25a	0.38	216.39abc	9.33	13.60cde	1.57	99.00b	9.17	7.13bc	0.20


*S1, rice tillering stage; S2, booting stage; S3, ripening stage; A, control treatment; B, NPK treatment; C, NPK + pig manure treatment; D, NPK + straw treatment; OM, organic matter; TN, total N; TP, total P; TK, total K; AN, available N; AP, available P; and AK, available K. Different letters in the same column indicate a significant difference (ANOVA followed by Tukey-Kramer post hoc test, n = 3, p < 0.05, average value, SD, standard deviation).*

**Table 2:** Effects of the sampling time and fertilization treatment on soil physicochemical properties (two-way ANOVA, ^∗^*p* < 0.05. ^∗∗^*p* < 0.01. ^∗∗∗^*p* < 0.001).

	OM	TN	TP	TK	AN	AP	AK	pH
Time	0.001**	0.126	0.000***	0.000***	0.000***	0.000***	0.000***	0.000***
Fertilizer	0.000***	0.000***	0.000***	0.067	0.003**	0.000***	0.012*	0.000***
Time ^∗^ Fertilizer	0.624	0.115	0.444	0.000***	0.025*	0.001**	0.331	0.015*


***Time,** Sampling time: rice tillering stage (S1), booting stage (S2), and ripening stage (S3). Fertilizer, Fertilization treatment: control treatment (A), NPK treatment (B), NPK + pig manure treatment (C), and NPK + straw treatment (D).*

### Bacterial α-Diversity

Bacterial richness and diversity indices were calculated based on randomly selected subset of 47,847 sequences per soil sample. Significant differences (*p* < 0.05) in the Ace, Chao1, Shannon, and Simpson indices were found among the different sampling times (Supplementary Table S1), but no significant difference in Ace and Chao1 was found among the different fertilization treatments and rice growth stages. The Shannon index was also not significantly different between the four fertilizer treatments and the three stages. Significant differences in the Simpson index were found among the S2C, S1B, S1C, and S1D samples, but no significant difference was found among the four fertilizer treatments at the same growth stage. The bacterial α-diversity indices were not correlated with the four fertilization treatments and the three rice growth stages.

### Effects of the Rice Growth Stage and Fertilizer Treatment on Soil Bacterial Communities and Bacterial Functions

**FIGURE 1:**
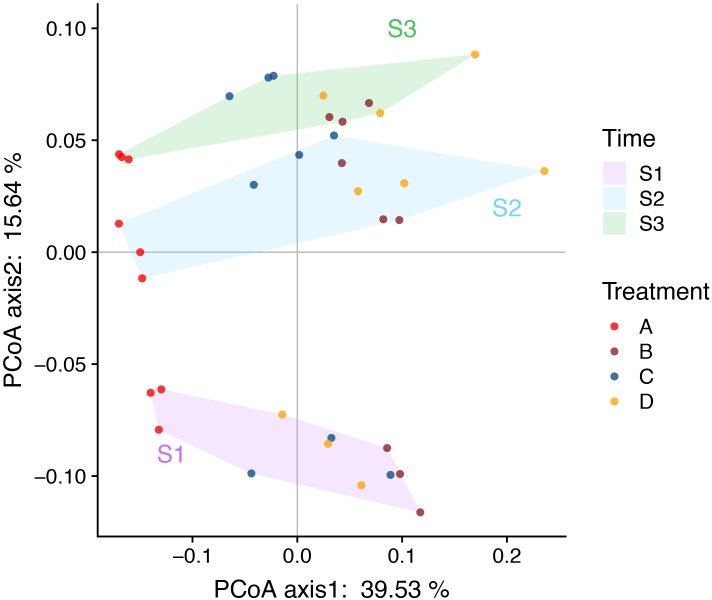
PCoA (Bray-Curtis distance index) plots allowing visualization of the differences in the bacterial community structure between samples (based on OTU information). The background color of the sample names indicates the sampling time: purple indicates the S1 stage, nattier blue indicates the S2 stage, and grass green indicates the S3 stage. The color of the sample points indicates the four fertilizers treatments: red indicate the control treatment (A); brown indicate the NPK treatment (B); blue indicate the NPK + pig manure treatment (C), and orange indicate the NPK + straw treatment (D). The soil samples were arranged left-to-right based on the four fertilizers treatments and top-to-bottom based on the three rice growth stages (the later stages are found at the top).

The PCoA and ANOSIM results showed that the bacterial community similarity distance was influenced by the fertilization treatment and rice growth stage (Figure 1 and Tables 3, 4). The samples from the four fertilizer treatments were divided by the PCoA axis1 (Figure 1). In particular, the control treatment without fertilizers (A) was quite different from the other three fertilization groups, as also shown by ANOSIM analysis (*p* < 0.05, Table 3). In addition, based on the PCoA axis1, the C treatment (NPK plus pig manure) differed from the B (NPK) and D (NPK plus straw) treatments at the S2 and S3 stages (Figure 1). However, no distinction was found between the B (NPK) and D (NPK plus straw) treatments (*p* > 0.05, Table 3). In contrast, the influence of the rice growth stages was mainly reflected in the PCoA axis2. The samples collected at S1 were distinguishable from those collected at S2 and S3, and ANOSIM analysis showed that the separation was still significant (Table 4, *p* < 0.01). In addition, the COG_L2 (Supplementary Figure S1A) and KEGG_L3 (Supplementary Figure S1B) profiles that were generated with the bacterial 16S rRNA gene results were used for the PCoA analysis of soil bacterial function. The results showed that the soil bacterial function could be separated among the S1 and other two stages but not the four fertilization treatments (Supplementary Figure S1).

**Table 3:** ANOSIM analysis between four fertilizer treatments.

	A: control		B: NPK		C: NPK + pig manure		D: NPK + straw	
	*R*	*p*	*R*	*p*	*R*	*p*	*R*	*p*
A: control			**0.985**	0.001***	**0.771**	0.001***	**0.826**	0.002**
B: NPK	0.659	0.002**			**0.344**	0.006**	**0.057**	**0.212**
C: NPK + pig manure	0.446	0.001***	0.159	0.029*			**0.057**	**0.197**
D: NPK + straw	0.706	0.001***	0.013	0.351	0.076	0.125		


Analysis of similarity was calculated between all treatments based on OTUs tables Euclidean (regular font) and Bray-Curtis (bold font) distance matrices. Each pairwise comparison of two groups was performed using 999 permutations. R values > 0.75 are generally interpreted as clearly separated, R > 0.5 as separated and R < 0.25 as groups hardly separated (Krych et al., 2013). *p < 0.05, **p < 0.01, ***p ≤ 0.001.

**Table 4:** ANOSIM analysis between rice growth stages.

	S1		S2		S3	
	*R*	*p*	*R*	*p*	*R*	*p*
S1			**0.31**	0.002**	**0.475**	0.001***
S2	0.213	0.002**			**0.097**	**0.067**
S3	0.41	0.001**	0.054	0.116		


Analysis of similarity was calculated between all stages based on OTUs tables Euclidean (regular font) and Bray-Curtis (bold font) distance matrices. Each pairwise comparison of two groups was performed using 999 permutations. R values > 0.75 are generally interpreted as clearly separated, R > 0.5 as separated and R < 0.25 as groups hardly separated (Krych et al., 2013). And the sampling time refers to the rice tillering stage (S1), booting stage (S2), and ripening stage (S3). *p < 0.05, **p < 0.01, ***p ≤ 0.001.

### Correlations of Soil Properties With Soil Bacterial Communities and Functions

**FIGURE 2:**
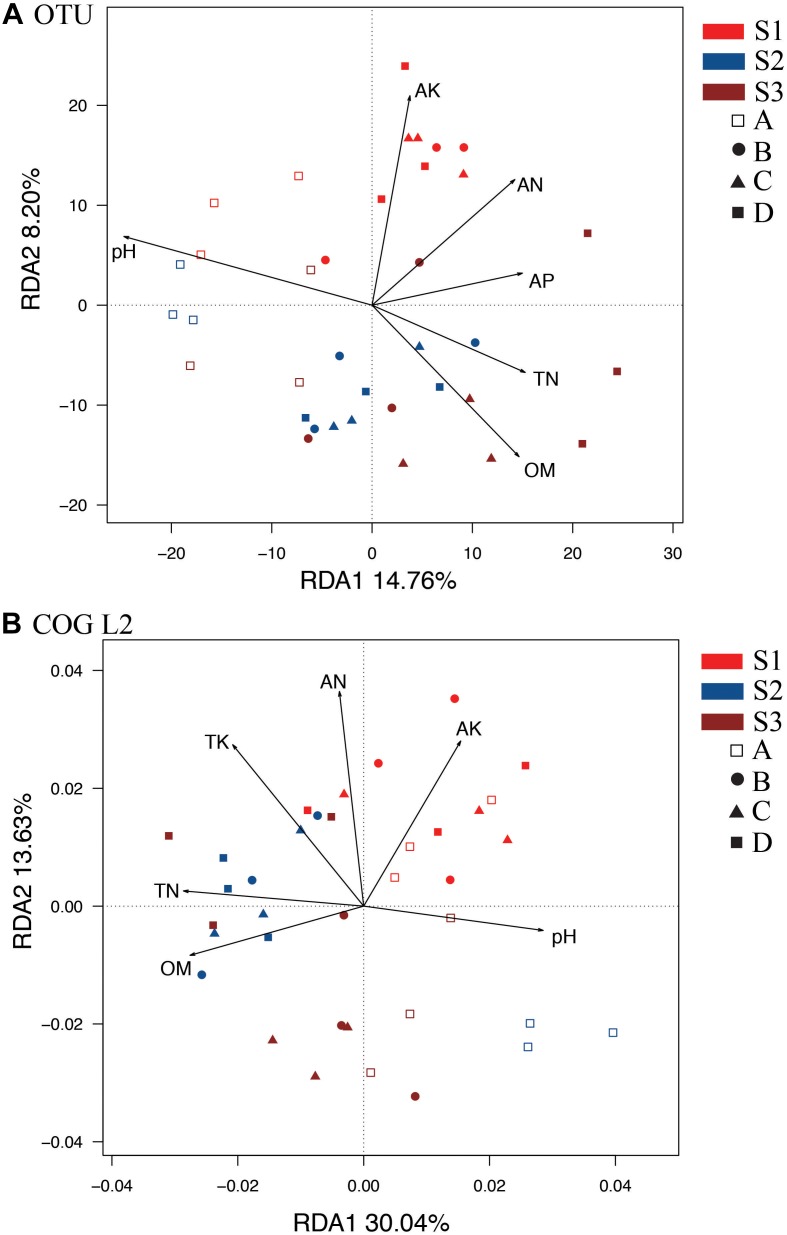
RDAs between soil samples at different stages based on OTU **(A)** and COG_L2 **(B)** levels. The colors of the sample names indicate the sampling time: red indicates the S1 stage, brown indicates the S2 stage, and blue indicates the S3 stage. The hollow squares indicate the control treatment (A); the circles indicate the NPK treatment (B); the triangles indicate the NPK + pig manure treatment (C), and the solid squares indicate the NPK + straw treatment (D). OM, organic matter; TN, total N; TP, total P; TK, total K; AN, available N; AP, available P; AK, available K.

We performed a redundancy analysis (RDA) to understand the OTU-level bacterial community structure (Figure 2). Six parameters, namely, pH, OM, TN, AN, AP, and AK (Supplementary Table S2), were selected based on variance inflation factors with 999 Monte Carlo permutations. The *p*-values obtained for OM, AK, and pH were significantly lower than those of TP and TK. These six environmental variables together explained 31.13% of the total variance. In addition, the first axis of the RDA accounted for a higher variation of 14.76%, whereas the second axis accounted for a lower variation of 8.20% (Figure 2A). The A treatment (control without fertilizers) could be distinguished from the other treatments based on the direction of change in the pH, OM and TN. However, S1 was distinguished from S2 and S3 by the direction of changes in AK and AN. Variance partitioning analyses (VPA) were further performed to assess the contributions of soil pH and nutrients to the bacterial community variances. The results showed that the pH alone could explain the higher variation of 6.42%, whereas the OM, AK, TN, and AP contents explained lower variations of 3.05, 2.97, 2.38, 2.17, and 2.39%, respectively. These results suggested that pH, OM, and AK appeared to be key factors responsible for the microbial community changes observed in all the treatments. In addition, the RDA of bacterial function (Figure 2B, COG L2) also showed that S1 was distinct from S2 and S3 based on the different directions of change in the AK and AN. The control treatment could be distinguished from the others by the direction of change in the pH, OM, and TN (Figure 2B).

### Correlation Between the Variation in the Abundance of Soil Bacteria and Bacterial Function During the S1 and S2/S3 Stages

**FIGURE 3:**
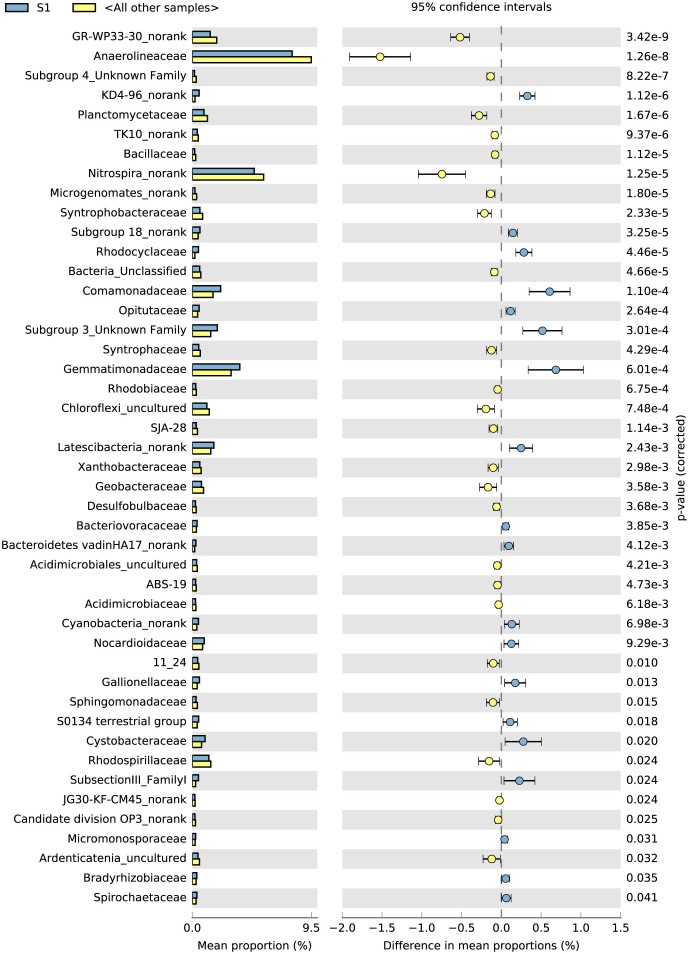
Top 100 microorganism families during the different sampling times identified using STAMP. The different colors of the oblong blocks indicate the different rice growth stages. The blue blocks indicate the rice tillering stage (S1), and the yellow blocks indicate all the other samples (S2 and S3 stages) (*p* < 0.05, average proportion, *n* = 3).

The top 100 bacterial families found at the highest abundances were used to evaluate the effects of the rice phonological stage and fertilizer treatment on the bacterial community. The STAMP results (average abundance, *n* = 3, *p* < 0.05) showed that the top 100 families exhibited significant differences between S1 and the other stages (Figure 3). From S1 to S3, the abundances of *Anaerolineaceae*, *Desulfobulbaceae*, *Planctomycetaceae*, *Rhodospirillaceae*, and *Geobacteraceae* increased, whereas those of *Comamonadaceae*, *Opitutaceae*, *Gemmatimonadaceae*, *Bacteriovoracaceae*, *Nocardioidaceae*, *Gaiellaceae*, and *Cystobacteraceae* decreased. In addition, compared with the control without fertilizers (A), the application of NPK fertilizer significantly influenced the bacterial family community (Supplementary Figure S2). *Nitrospinaceae*, *Nocardioidaceae*, *Nitrosomonadaceae*, *Cytophagaceae*, and *Gaiellaceae* were found at high abundances in the control treatment (A), whereas *Acidobacteriaceae*, *Xanthomonadaceae*, *Cystobacteraceae*, *Gemmatimonadaceae*, and *Geobacteraceae* were enriched in the NPK fertilizer treatments (B, C, and D).

The KEGG (Kyoto Encyclopaedia of Genes and Genomes) and COG metabolic pathways of soil bacteria were analyzed using PICRUSt. The results for metabolic functions were similar to the bacterial abundance results. Specifically, both the KEGG and COG metabolic pathways exhibited significant differences among the rice growth stages in the control without fertilizers (A) and the chemical fertilizer treatments (Supplementary Figures S3, S4). The results of the COG_L2 functional difference showed that there was a significant functional difference between S1 and S2 and S3 (Supplementary Figure S3, *P* < 0.05). Such as, [A] RNA processing and modification, [C] Energy production and conversion, [I] Lipid transport and metabolism, [K] Transcription, etc. For the fertilizer treatment, the control without fertilizers (A) was significantly different from the other three treatments in 15 functional classifications (Supplementary Figure S3, *p* < 0.05). Such as, [I] Lipid transport and metabolism, [E] Amino acid transport, and metabolism, [H] Coenzyme transport and metabolism, etc. (Supplementary Figure S3). There were no significant functional metabolic differences between B and D treatments, and only [A] RNA processing and modification differed between C and D treatments (Supplementary Figure S3, *P* < 0.05).

The results of the KEGG_L2 functional difference showed that S1 and S2 and S3 has a significant (*P* < 0.05) difference of nearly 21 functional classifications, including Energy Metabolism, Amino Acid Metabolism, Carbohydrate Metabolism, Membrane Transport like, etc. (Supplementary Figure S4, *p* < 0.05). But there were only 5 significant functional classifications between the S2 and S3 groups (Supplementary Figure S4, *P* < 0.05). For the fertilizer treatment, the control without fertilizers (A) was significantly different from the other three treatments in 14 functional classifications (Supplementary Figure S4, *p* < 0.05). Such as, Lipid Metabolism, Amino Acid Metabolism and Glycan Biosynthesis and Metabolism like etc. There were 4 functional classifications differences between B and C treatments, while there was no significant difference between the others treatments (Supplementary Figure S4).

The correlations among the differentiated bacterial families, soil properties and KEGG metabolic pathways are illustrated in Supplementary Table S3 and plotted in Supplementary Figure S5 (Ma et al., 2014). The abundances of bacterial families, such as *Micromonosporaceae, Nocardioidaceae, Gaiellaceae, Anaerolineaceae, Syntrophobacteraceae*, and *Syntrophaceae*, were correlated with bacterial KEGG metabolic pathways. Specifically, 70–80% of the identified bacterial families were significantly correlated with amino acid metabolism (Such as Alanine aspartate and glutamate metabolism, Tryptophan metabolism, Phenylalanine tyrosine, and tryptophan biosynthesis, Tyrosine metabolism, etc.), the biosynthesis of other secondary metabolites (Isoquinoline alkaloid biosynthesis, beta-Lactam resistance, Flavone and flavonol biosynthesis, etc.), carbohydrate metabolism (Citrate cycle (TCA cycle), Butanoate metabolism, Propanoate metabolism, etc.), glycan biosynthesis and lipid metabolic pathways (Protein kinases, Peptidoglycan biosynthesis, Glycosaminoglycan degradation, etc.) (Supplementary Figure S5). We also used the Spearman’s rank-order correlation method to evaluate the relationships between KEGG metabolic pathways and soil properties (Supplementary Figure S5) and found that the soil TK, AN, pH, and AK were significantly correlated with most metabolic pathways or differentiated bacterial families. In addition, the differentiated families were also significantly correlated with the primary soil properties, such as pH, AK, and AN (Supplementary Figure S6). These results showed that the soil properties, differentiated bacterial families and KEGG metabolic pathways were closely related to each other.

### Molecular Ecology Network Analysis of Soil Bacterial Communities and Functions at the Different Rice Growth Stages

**FIGURE 4:**
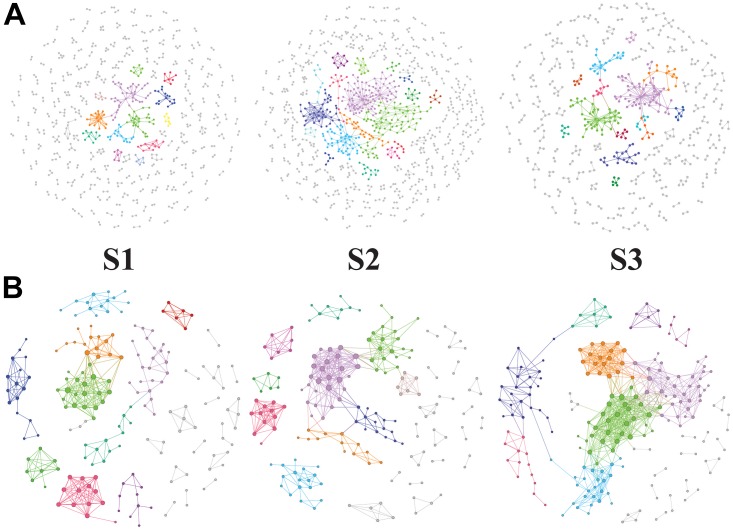
Succession of soil bacteria (**A**, OTU level) and bacterial metabolic pathway (**B**, KEGG L3 produced by PICRUSt) networks during the different stages of rice growth (tillering, heading and ripening). The networks represent the random matrix theory co-occurrence models derived from 12 biological replicates at each time point. In the models, the nodes represent the OTUs or KEGG L3 metabolic pathways. The red links indicate significant positive correlations between two individual nodes, whereas the blue links indicate negative covariations. The modules are randomly colored at each growth stage, and the nodes in the modules with less than six nodes are shown in black.

The potential bacteria-bacteria interactions were analyzed using bacterial co-occurrence networks (Deng et al., 2012). Both the network size and the degree of connectivity showed differences among the rice growth stages. At the S2 and S3 stages, the bacterial assemblages formed larger and more complex network topological structures, with increasing numbers of links and nodes, compared with the S1 stage networks (Figure 4 and Table 5). The S1 networks were smaller than the others. The increased numbers of nodes in the S2 network were connected and formed larger modules, whereas the size of each module in the network of the S3 stage was larger than that of the S1 network and smaller than that of the S2 networks. The increased complexity of the S2 networks displayed an average degree of increase and shorter harmonic geodesic distances (HD) (Table 5; Deng et al., 2012).

**Table 5:** Global properties of the networks based on OTUs and KEGG_L3.

Network indices	OTUs (0.950)			KEGG_L3 (0.930)		
	S1	S2	S3	S1	S2	S3
Total nodes	682	931	823	187	185	209
Total links	558	959	884	393	452	789
R square of power-law	0.95	0.88	0.96	0.85	0.79	0.72
Average degree (avgK)	1.64	2.06	2.15	4.20	4.89	7.55
Average clustering coefficient (avgCC)	0.04	0.06	0.07	0.49	0.52	0.52
Average path distance (GD)	0.13	0.81	0.28	3.58	4.08	6.41
Geodesic efficiency (E)	0.01	0.02	0.02	0.45	0.36	0.26
Harmonic geodesic distance (HD)	146.24	53.97	67.93	2.24	2.79	3.88
Maximal degree	13	46	36	17	21	30


*Average connectivity (avgK), a higher avgK indicates a more complex network; the average clustering coefficient (avgCC) is used to measure the extent of module structure present in a network; average path distance (GD), a smaller GD indicates that all the nodes in the network are closer; the geodesic efficiency (E) is the opposite of GD, i.e., a higher E indicates that the nodes are closer; harmonic geodesic distance (HD), the reciprocal of E, which is similar to GD but more appropriate for a disjointed graph; and the sampling time refers to the rice tillering stage (S1), booting stage (S2), and ripening stage (S3).*

We also used the KEGG L3 metabolic pathway profile to construct bacterial functional networks for three rice growth stages. The bacterial functions exhibited denser connections at the KEGG L3 level. The S1 and S2 stages exhibited more similar network topological structures, but analyses of the average degree (avgK), average clustering coefficient (avgCC), and average path distance (GD) in all three networks revealed that the S2 network contained 20% more links than the S1 (Table 5). The structure of the S2 network is more complex than that of the S1 network (with higher avgK, a higher avgK indicates a more complex network). The number of nodes and links and the values of other network indices obtained for the S3 network were clearly higher than those found for the S1 and S2 networks. At the S3 stage, a greater number of bacterial functional pathways were connected and formed an intimate yet complicated co-occurrence network.

## Discussion

### Effects of Treatments and Rice Stage on the Soil Chemical Properties

The soil bacterial communities are commonly influenced by the availability of soil nutrients and their ability to absorb these limited nutrients (Noll et al., 2005). The soil nutrient status could be significantly affected by different chemical and organic fertilizer treatments. In this study, the soil nutrient status showed distinct changes among different stages of rice phonology. Similarly, the soil physical and chemical gradients are dependent on plant development over time (Krause et al., 2010). Some key soil properties, such as pH, AK, OM, and AN, were significantly affected by the fertilizer treatments and rice growth stages, and previous studies showed that pH, AK, OM, and AN are critical factors that influence the soil bacterial communities (Anderson et al., 2014; Wu et al., 2014; Sun et al., 2015; Zhou et al., 2015; Ding et al., 2016; Li et al., 2017). The changes in soil properties among different rice growth stages is likely due to the effects of the slow release of fertilizer, the uptake of nutrients by the plant and various root secretions (an effect on bulk soil mediated by secretions looks like a longshot, e.g., organic acids, amino acids, sugars, and growth substances) (Huang et al., 2014; Reinhold-Hurek et al., 2015).

### Effects of Treatments and Rice Stages on the Soil Bacterial Community Composition

Although the bacterial α-diversity indices, such as Ace, Chao1, Shannon, and Simpson, were not significantly different (*p* < 0.05) among different fertilizer treatments and rice growth stages, the bacterial beta-diversity (PCoA and RDA) results showed that the bacterial community showed significant differences among the fertilization treatments and rice stages. At all the stages, the bacterial community composition obtained with the A treatment (control) was quite different from that obtained with the other three fertilization groups, and the B (NPK) and D (NPK + straw) treatments were grouped together. The inclusion of pig manure in the C treatment improved the bacterial community composition at the S2 and S3 stages. These results strengthened the notion that the amendment of livestock manure could prevent the negative effects of the long-term application of chemical fertilizers on the bacterial community in a field (Sun et al., 2015), such as the decrease in the number of microorganisms, the decrease in the diversity index, and the decrease in the abundance of beneficial microorganisms such as *rhizobium* and *myxobacteria* (Wang et al., 2016c). In this study, pig manure exerted stable effects on the bacterial community that appeared to persist throughout all rice stages.

Yu suggested that studies of the bacterial community should pay more attention to different stages other than fertilization (Yu et al., 2015). In this study, we selected three critical rice stages and found significant differences in the bacterial community. The succession of the bacterial community from the early growth stage (S1) to S2 and S3 could be clearly distinguished, whereas bacterial succession became relatively stable at the S2 and S3 stages. The finding that the soil bacterial community was relatively stable at the middle and later stages was also confirmed by PLFA analyses of a rice field (Noll et al., 2005). The researchers noted that aerobic and anaerobic conversion caused significant changes in the bacterial community at the early rice growth stages; specifically, the bacterial community gradually transitioned from the early to the late community structure at the middle stage but not at the late stage (Noll et al., 2005). Simultaneously, the differences between the bacterial community and functional gene succession processes at different stages (or sampling times) were also found in rice (Rui et al., 2009), wheat (Jing et al., 2015), maize (Li et al., 2014), nitrogen-fixing organisms (Wang et al., 2016b), and methanotrophs (Krause et al., 2010).

### Growth-Stage Related Dynamics of the Soil Bacterial Function Community Structures

Soil bacterial functional genes show changes among the treatments and rice stages (Langille et al., 2013). The PCoA, RDA, and STAMP results indicated that the bacterial functional gene communities were affected by both the fertilization treatments and the rice developmental stages. We found an obvious succession of the bacterial functional community during rice development. In addition, the NPK fertilizers had significant effects on bacterial metabolic functions. Previous studies have revealed that higher-quality organic fertilizer management could improve the soil bacterial functional community (Guan et al., 2011; Yu-Hong et al., 2011). The bacterial functional structures at the S1 stage were significantly different from those at the S2 and S3 stages, which presented relatively stable bacterial functional structures. Other studies revealed a similar effect in the *nif*H gene (Yuan et al., 2015; Wang et al., 2016b), carboxylic acids (Yu et al., 2015), and anammox rate (Li et al., 2016). It is likely that the combination of physical (root structure characteristics) and chemical (root exudation) changes contributes to the succession of the bacterial functional structure during rice development (Yuan et al., 2015). In addition, the RDA results also proved that the soil available nutrients AN and AK appeared to be key factors in the succession of the bacterial function community.

In addition, based on the correlations between the most abundant differentiated bacterial families and KEGG metabolic pathways and between soil properties and KEGG metabolic pathways, we concluded that there is a significant correlation among bacterial composition, KEGG metabolic pathways and soil properties. The succession of the bacterial compositions, KEGG metabolic pathways and soil properties occurred nearly concurrently with the progression through the rice developmental stages.

### Molecular Ecology Network Analysis of Soil Bacterial Communities and Functions at Different Rice Growth Stages

Molecular ecology network analysis has been widely used to explore the interactions between soil microorganisms and function genes (Zhou et al., 2010, 2011; Ren et al., 2015), and the results of these analyses have helped us understand the dynamic changes in microbial niches or function genes (Shi et al., 2016). In addition, recent studies have indicated that a network analysis of bacterial communities might allow the assessment of soil ecology or their contribution to habitat niches (Weiss et al., 2016). Compared with that at the early stage, the network structures at the S2 and S3 stages generated from the bacterial and KEGG function assemblages exhibited increased complexity. The interactions between bacteria or their KEGG functions varied during different plant growth stages. The same phenomenon has also been detected in vegetative rhizosphere soil (Shi et al., 2016), and changes in network complexity have even been found between fallow and mature periods in tobacco fields (Niu et al., 2016), between different seasons in eutrophic Lake Mendota (Kara et al., 2013), and between different years in salt marsh chronosequence (Dini-Andreote et al., 2014). The succession of networks during rice growth was reflected in the soil chemical properties and bacterial community changes.

The key question addressed in this study is the primary factor driving the succession of the network, and the quality and quantity of root exudates might be this factor because organic acids show changes among different rice phonological stages (Li et al., 2016). In addition, significantly lower root exudates are found in bulk soil (Li et al., 2016). Previous research suggests that roots promote the development of soil microbial niches, which results in interactions and covariations among the bacteria (Shi et al., 2016). The differences in temperatures, precipitation and sunlight hours among different stages might be responsible for the differences in the growth characteristics of cultivated bacteria. Climate change has altered plant phenology and microbial communities (Classen et al., 2015).

However, the network of functional genes, unlike the bacterial network, at the S3 stage exhibits a more complex (higher avgK) network organization than those at the S1 and S2 stages. The succession of the soil bacterial community network structure was only partially consistent with the changes in function. A more complex soil bacterial community network was formed at the middle stage of rice development, and the topological of the community network maintained a relatively similar structure (the parameters of the community network are nearly consistent) during the middle and late stages. It is worth noting that the topological of the functional network structure in the early and middle stages is more similar, but formed a complicated co-occurrence network at the S3 stage. The topological of functional network structure of bacteria changes a little in the early and middle stages of rice, while its changes significantly in the ripening stage of rice growth. It is possible that the topological of functional networks are closely related to the rice maturity stage. In addition, during rice growth, the formation of complex bacterial functional networks occurred later than the formation of complex bacterial networks, although changes in the microbial structure and functions are commonly observed under different environmental conditions (Mäder et al., 2002; Graham et al., 2014; Riah-Anglet et al., 2015), and the topological of changes in soil ecology function were not consistent with those in the community composition in a rice paddy (Chen et al., 2015). As described by Fry, the function of a grassland ecosystem is influenced by the experimental treatments but is not related to associated changes in the microbial community (Fry et al., 2016).

## Author Contributions

WW performed all the experiments, coordinated the data analyses, and prepared the manuscript. XL, YC, and XY contributed to the preparation of the manuscript and data analyses. ZheC and WR conceived the research study. ZLC and HW supervised the entire study.

## Conflict of Interest Statement

The authors declare that the research was conducted in the absence of any commercial or financial relationships that could be construed as a potential conflict of interest.

## Footnotes

1 http://qiime.org/

2 http://ieg2.ou.edu/MENA/
