# Supplementary material for: Succession of Composition and Function of Soil Bacterial Communities During Key Rice Growth Stages
Source: Front Microbiol. 2019 Mar 11;10:421. doi: 10.3389/fmicb.2019.00421 (PMC6422105; doi:10.3389/fmicb.2019.00421)
Supplement: Supplementary file 1 [file Data_Sheet_1.docx]

**Title Page**

**Succession of composition and function of soil bacterial communities during key rice growth stages**

Wenhui Wang^1,2^, Xue Luo^2^, Yang Chen^2^, Xianfeng Ye^2^, Hui Wang^1*^, Zhe Cao^3^, Wei Ran^4^ and Zhongli Cui^2*^

^1^Key Laboratory of Soil Environment and Pollution Remediation, Institute of Soil Science, Chinese Academy of Sciences, Nanjing, China

^2^Key Laboratory of Agricultural Environmental Microbiology of the Ministry of Agriculture, Nanjing Agricultural University, Nanjing, China

^3^Crop Development Centre/Department of Plant Sciences, University of Saskatchewan, Saskatoon, Canada.

^4^College of Resources & Environmental Sciences, Nanjing Agricultural University, Nanjing, China.

**Running Title:** Soil microbial community/functional structure

***Corresponding author**: Hui Wang

**Corresponding address:** Key Laboratory of Soil Environment and Pollution Remediation, Institute of Soil Science, Chinese Academy of Sciences, East Beijing Road 71, Nanjing 210008, China

**Tel.:** +862586881356, **Fax:** +8602586881000, **E-mail:** [hwang@issas.ac.cn](mailto:hychu@issas.ac.cn)

***Corresponding author**: Zhongli Cui

**Corresponding address:** Key Laboratory of Agricultural Environmental Microbiology of the Ministry of Agriculture, Nanjing Agricultural University, Nanjing 210095, China

**Tel.:** +8602584396753, **Fax:** +8602584396753, **E-mail:** [labc_7021@163.com](mailto:labc_7021@163.com); czl@njau.edu.cn

**Supplementary information I**

**Supplementary Figures and Tables**

**Table S1**

Table S1 Estimated richness and diversity indices of the different OTUs in the different stages of paddy fields (p < 0.05, average value, n= 3, SD = standard deviation).

| **Field** | | **Ace** | | **Chao1** | | **Shannon** | | **Simpson** | |
| --- | --- | --- | --- | --- | --- | --- | --- | --- | --- |
|  |  | Mean | SD | Mean | SD | Mean | SD | Mean | SD |
| **S1** | A | 6605 a | 57 | 6612 a | 82 | 7.20 ab | 0.03 | 2.27E-03 abcd | 1.19E-04 |
|  | B | 6478 a | 199 | 6496 a | 221 | 7.24 ab | 0.02 | 1.90E-03 cd | 6.85E-05 |
|  | C | 6489 a | 128 | 6468 a | 95 | 7.24 ab | 0.02 | 1.93E-03 cd | 4.82E-05 |
|  | D | 6635 a | 111 | 6583 a | 86 | 7.27 a | 0.02 | 1.85E-03 d | 7.61E-05 |
| **S2** | A | 6554 a | 141 | 6544 a | 88 | 7.19 ab | 0.05 | 2.28E-03 abc | 1.47E-04 |
|  | B | 6677 a | 113 | 6677 a | 148 | 7.22 ab | 0.06 | 2.31E-03 abc | 4.46E-04 |
|  | C | 6703 a | 152 | 6667 a | 291 | 7.18 ab | 0.07 | 2.54E-03 a | 5.53E-04 |
|  | D | 6491 a | 413 | 6442 a | 416 | 7.16 b | 0.08 | 2.15E-03 abcd | 1.36E-04 |
| **S3** | A | 6477 a | 111 | 6500 a | 183 | 7.16 b | 0.02 | 2.39E-03 ab | 8.06E-05 |
|  | B | 6684 a | 230 | 6597 a | 189 | 7.18 ab | 0.04 | 2.24E-03 abcd | 7.71E-05 |
|  | C | 6680 a | 157 | 6617 a | 143 | 7.20 ab | 0.03 | 2.29E-03 abc | 1.15E-04 |
|  | D | 6670 a | 231 | 6628 a | 256 | 7.24 ab | 0.06 | 2.06E-03 bcd | 6.06E-05 |

Note: Different letters in the same column indicate a significant difference (p < 0.05). S1 indicates the rice tillering stage; S2 indicates the booting stage, and S3 indicates the ripening stage. A: the control treatment, B: the NPK treatment, C: the NPK + pig manure treatment and D: the NPK + straw treatment.

**Table S2**

Table S2 The Monte Carlo permutation test

|  | RDA1 | RDA2 | r2 | Pr(>r) |  |
| --- | --- | --- | --- | --- | --- |
| OM | 0.52538 | -0.85087 | 0.3640 | 0.001 | *** |
| TN | 0.84648 | -0.53242 | 0.2164 | 0.017 | * |
| TP | 0.82253 | 0.56872 | 0.1565 | 0.064 | . |
| TK | 0.58827 | 0.80867 | 0.0998 | 0.170 |  |
| AN | 0.75889 | 0.65122 | 0.3231 | 0.002 | ** |
| AP | 0.98405 | 0.17789 | 0.1799 | 0.034 | * |
| AK | 0.30626 | 0.95195 | 0.4212 | 0.001 | *** |
| pH | -0.89631 | 0.44342 | 0.5109 | 0.001 | *** |

OM: Organic matter, TN: Total N, TP: Total P, TK: Total K, AN: Available N, AP: Available P, AK: Available K.

Signif. codes: 0 ‘***’ 0.001 ‘**’ 0.01 ‘*’ 0.05 ‘.’ 0.1 ‘ ’ 1

Permutation: free

Number of permutations: 999

**Table S3. Correlations between a family-level bacterial abundance, soil properties and PICRUSt-generated KEGG metabolic pathways profile.**

**Please find Table S3 in the supplement Excel file.**

**Figure S1.**


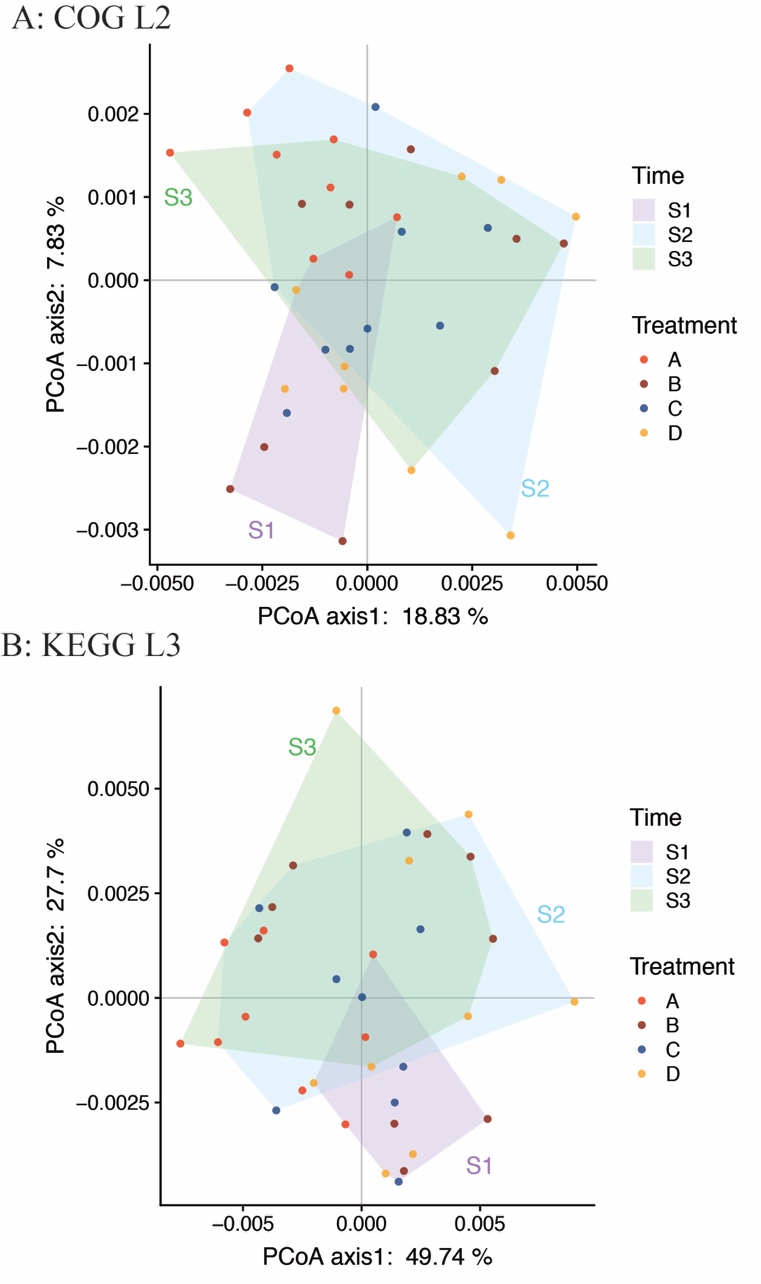


**Figure S1.** PCoAs between the different soil stages based on COG_L2 (A) and KEGG_L3 (B) level.

The background colour of the sample names indicates the sampling time: purple indicates the S1 stage, nattier blue indicates the S2 stage, and grass green indicates the S3 stage. The colour of the sample points indicates the four fertilizers treatments: red indicate the control treatment (A); brown indicate the NPK treatment (B); blue indicate the NPK + pig manure treatment (C), and orange indicate the NPK + straw treatment (D).

**Figure S2.**


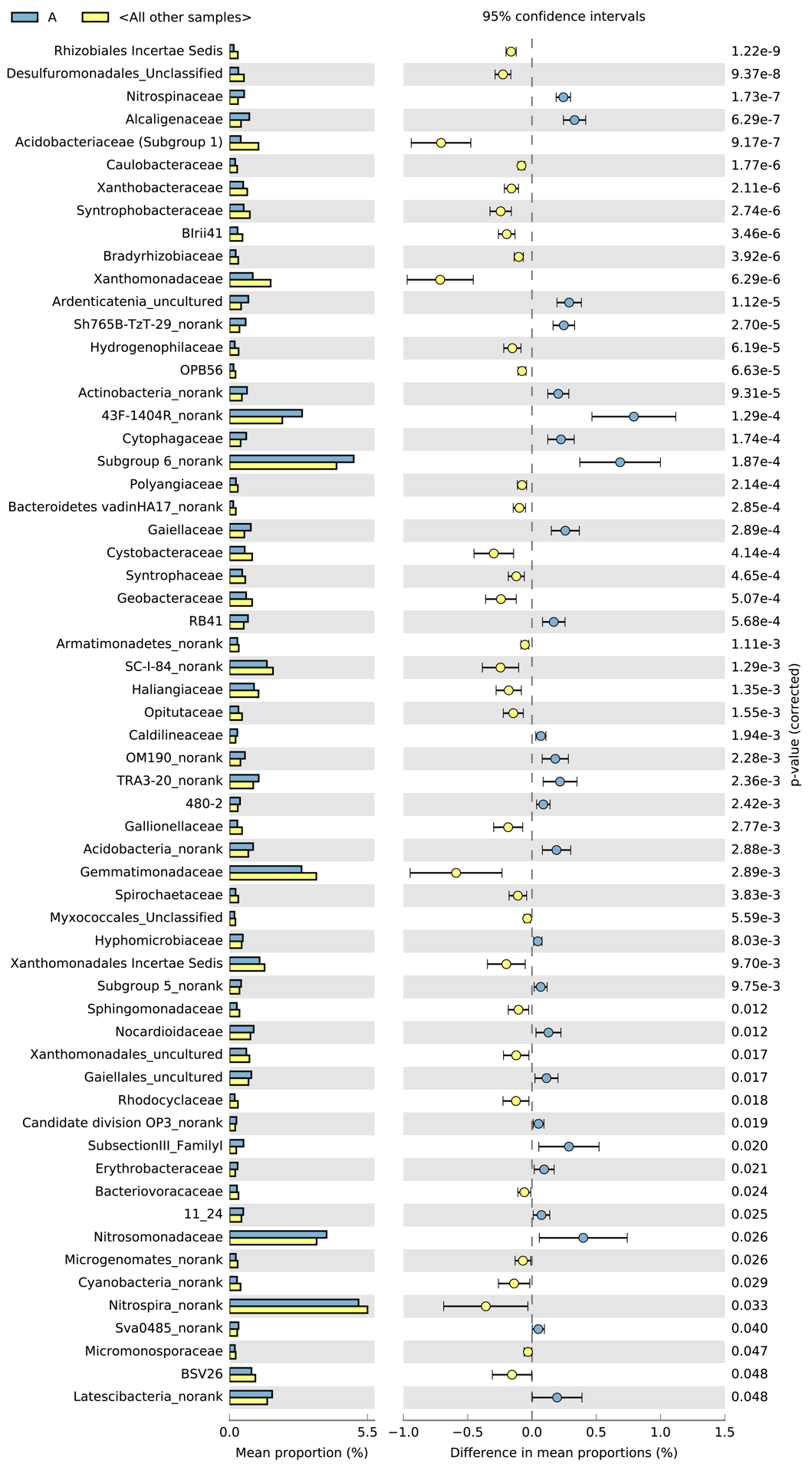


**Figure S2.** Top 100 microorganism families identified during the different fertilization treatments by STAMP.

Different colours of oblong blocks indicate different rice growth stages. The blue blocks indicate the control treatment (A), and the yellow blocks indicate all other samples (NPK treatment (B), NPK + pig manure treatment (C) and NPK + straw treatment (D)). (p < 0.05, average proportion, n = 3)

**Figure S3.**


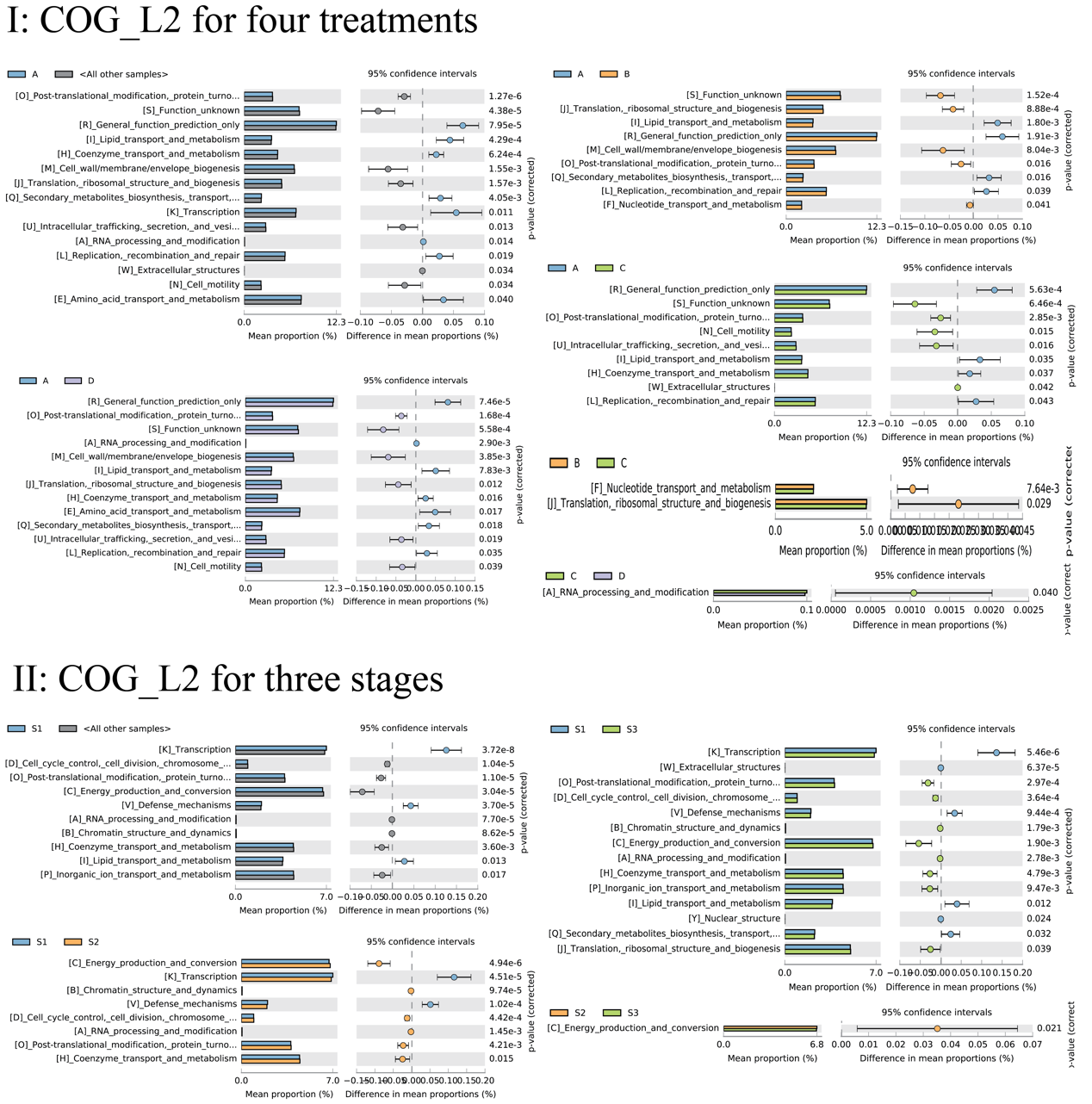


**Figure S3.** Different COG L2 function during different fertilization treatments (Ι) and rice stages (ΙΙ) by STAMP

(Ι) Different colours of oblong blocks indicate different rice growth stages. S1 indicates the rice tillering stage; S2 indicates the booting stage, and S3 indicates the ripening stage. All other samples indicate the S2 & S3 stages. (ΙΙ) Different colours of oblong blocks indicate different fertilizer treatments. A indicates the control treatment; B indicates the NPK treatment; C indicates the NPK + pig manure treatment, and D indicates the NPK + straw treatment. (p < 0.05, average proportion, n= 3)

**Figure S4.**

**
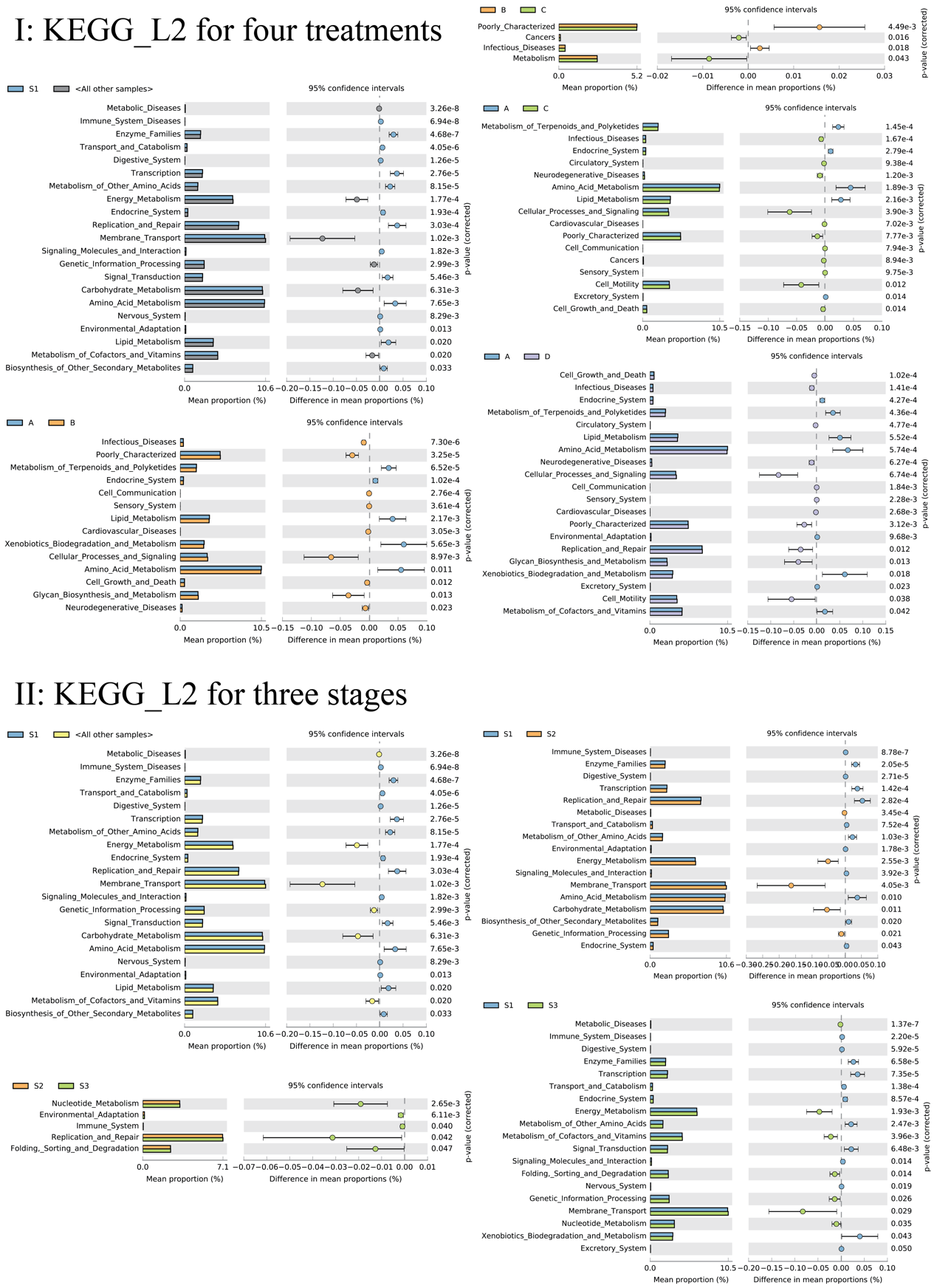
**

**Figure S4.** Different KEGG L2 function during different fertilization treatments (Ι) and rice stages (ΙΙ) by STAMP

(Ι) Different colours of oblong blocks indicate different rice growth stages. S1 indicates the rice tillering stage; S2 indicates the booting stage; S3 indicates the ripening stage, and all other samples indicate the S2&S3 stages. (ΙΙ) Different colours of oblong blocks indicate different fertilizer treatments. A indicates the control treatment; B indicates the NPK treatment; C indicates the NPK + pig manure treatment, and D indicates the NPK + straw treatment. (p < 0.05, average proportion, n= 3)

**Figure S5.**

**Please find Figure S5. in the supplement PDF file.**

**Figure S5.** Correlations between family-level bacterial abundance (forward selected by STAMP as being different between S1 and other two stages), soil properties and PICRUSt-generated KEGG metabolic pathways profile were plotted.

Metabolic pathway designations are indicated at the bottom of the figure. Size of the bubble indicates Spearman’s correlation coefficient between the family and KEGG pathway. Blue indicates a positive covariation between two individual nodes, while orange indicates a negative covariation. The coloured bubbles indicate significant correlations (Spearman’s; P<0.05), while the “blank” spaces indicate no significant correlation. OM: Organic matter, TN: Total N, TP: Total P, TK: Total K, AN: Available N, AP: Available P, AK: Available K.

**Figure S6.**


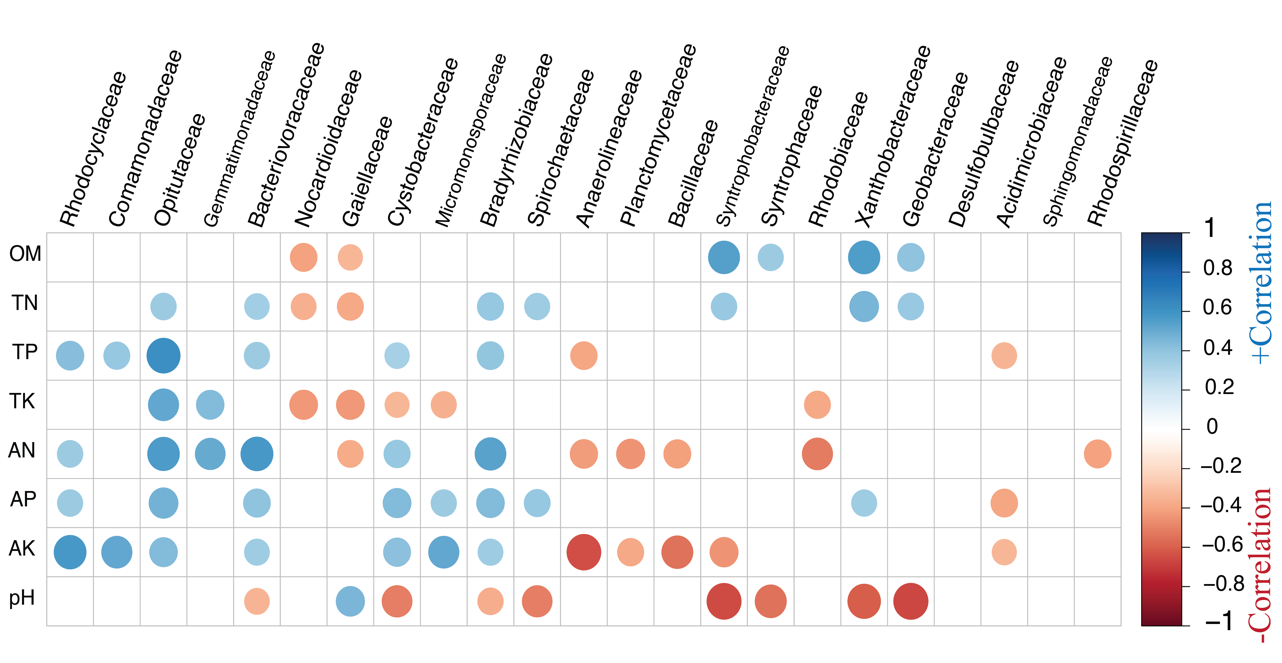


**Figure S6.** Spearman’s rank correlations (r) between bacterial family and soil properties

Size of the bubble indicates Spearman’s correlation coefficient between family and soil properties. Blue indicates a positive covariation between two individual factors, while orange indicates a negative covariation. The coloured bubbles indicate a significant correlation (Spearman’s; p < 0.05), while the “blank” spaces indicate no significant correlation.
