# Supplementary figures and images for: Succession of Composition and Function of Soil Bacterial Communities During Key Rice Growth Stages
Source: Front Microbiol. 2019 Mar 11;10:421. doi: 10.3389/fmicb.2019.00421 (PMC6422105; doi:10.3389/fmicb.2019.00421)

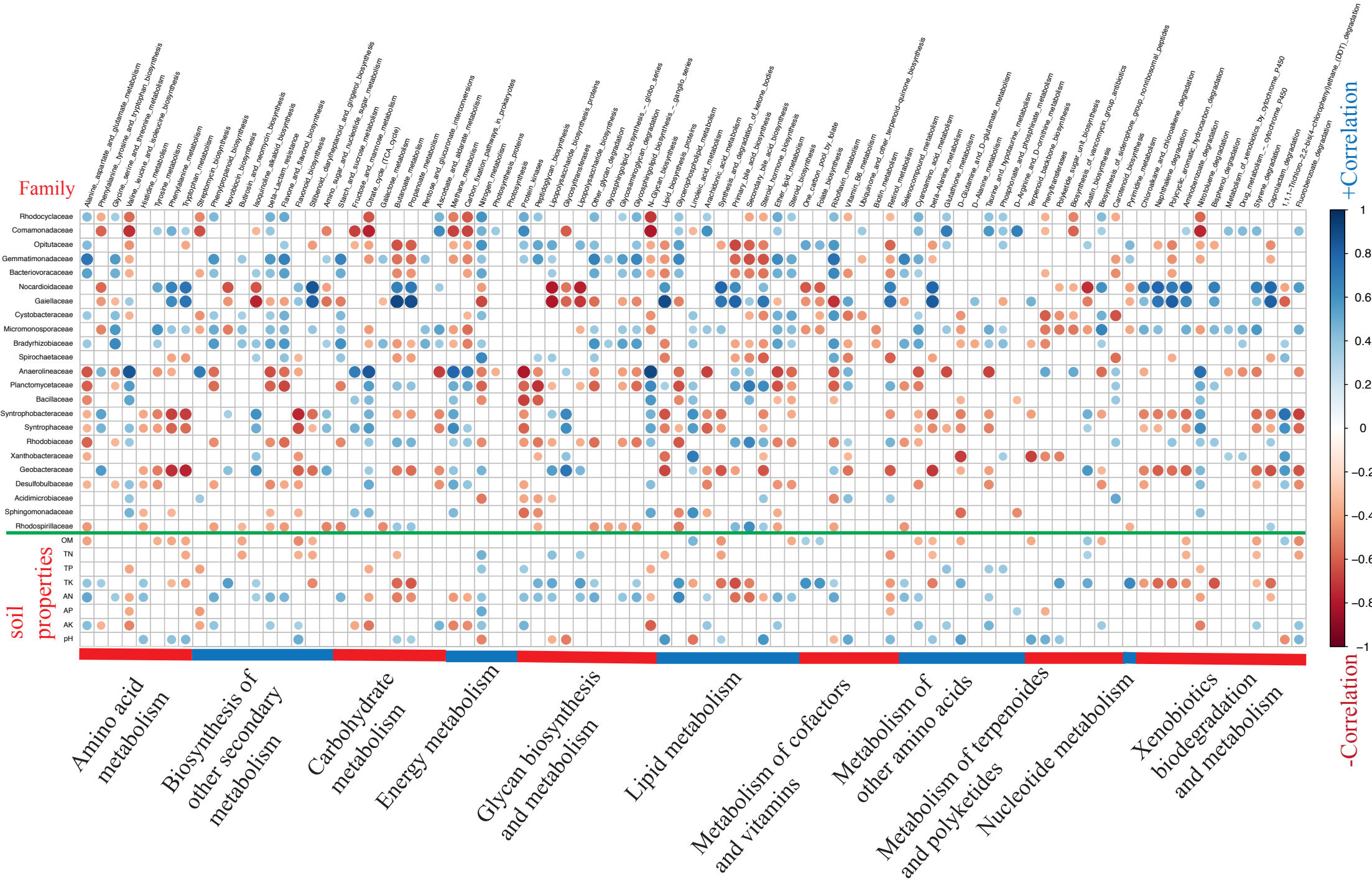

Supplement: Supplementary file 3 [file Data_Sheet_3.pdf]
